# Use of a transcervical approach to retrieve a foreign body from the upper esophagus

**DOI:** 10.1002/ccr3.9272

**Published:** 2024-08-06

**Authors:** Brihaspati Sigdel, Amrit Pokhrel, Bipin Subedi, Indra Subedi, Bidhan Ghimire, Shankar Paudel

**Affiliations:** ^1^ Department of Otolaryngology & Head and Neck Surgery Gandaki Medical College Pokhara Nepal; ^2^ Metrocity Hospital Pokhara Nepal; ^3^ Kaski Sewa Hospital Pokhara Nepal; ^4^ Department of Anaesthesia Pokhara Academy of Health Sciences Pokhara Nepal; ^5^ Department of Pediatrics Annapurna Children and Women's Hospital Pokhara Nepal; ^6^ Department of Pediatrics Gandaki Medical College Pokhara Nepal

**Keywords:** extramural, foreign body esophagus, rigid esophagoscopy, transcervical approach

## Abstract

Foreign bodies such as bone with a sharp end can penetrate the esophageal wall and lie extramurally. When a foreign body is not found on rigid oesophagoscopy, reassessing with imaging is important. The transcervical approach is a better alternative for such patients to remove the foreign bodies.

## Graphical abstract

Intraoperative visualization of foreign body at neck and after removal.

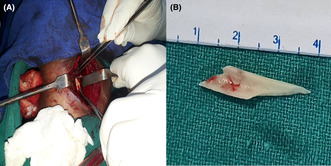

## 1 INTRODUCTION

The foreign body esophagus is a more common otorhinolaryngological emergency.^1^ It can occur in all age groups. However, it is common in extreme ages. The most common foreign bodies ingested are coins, marbles, fish bones, magnets, pins, candies, and meat bones. In most cases, it passes spontaneously. However, 1% or less cases require surgery.^2^ Among the surgical cases, ingestion of meat bone is common. In children, it becomes serious when a sharp bony foreign body is ingested compared to a coin. Timely, appropriate management is lifesaving in such types of cases. Rigid esophagoscopy is the method of choice for removal of the the foreign body in the esophagus. However, it cannot retrieve a foreign body that has pierced the digestive tract and migrated to extramural space. Here, we present a case of an extramural foreign body, which we removed via transcervical approach after failed endoscopic retrieval.

## 2 CASE REPORT

### 2.1 History

A 14‐year‐old male presented to the Annapurna Children and Women's Hospital with a 2‐day history of dysphagia and odynophagia. According to his mother, immediately after ingestion of goat meat at dinner, he complained of difficulty swallowing along with pain. So, they went to a nearby primary health center. Then, the patient was referred to the tertiary center at Pokhara. On arrival at the hospital, his blood pressure was 100/70 mmHg, pulse 94 beats/min, respiratory rate 20 breaths/min, temperature 98.8°F, and oxygen saturation (SPO2) 97% which were within normal range. He was treated with intravenous fluid; 1000 mL dextrose normal saline (DNS) and 500 mL Ringer Lactate (RL) over 24 h and intravenous ketorolac 15 mg four times in a day.

### 2.2 Investigation and treatment

**FIGURE 1:**
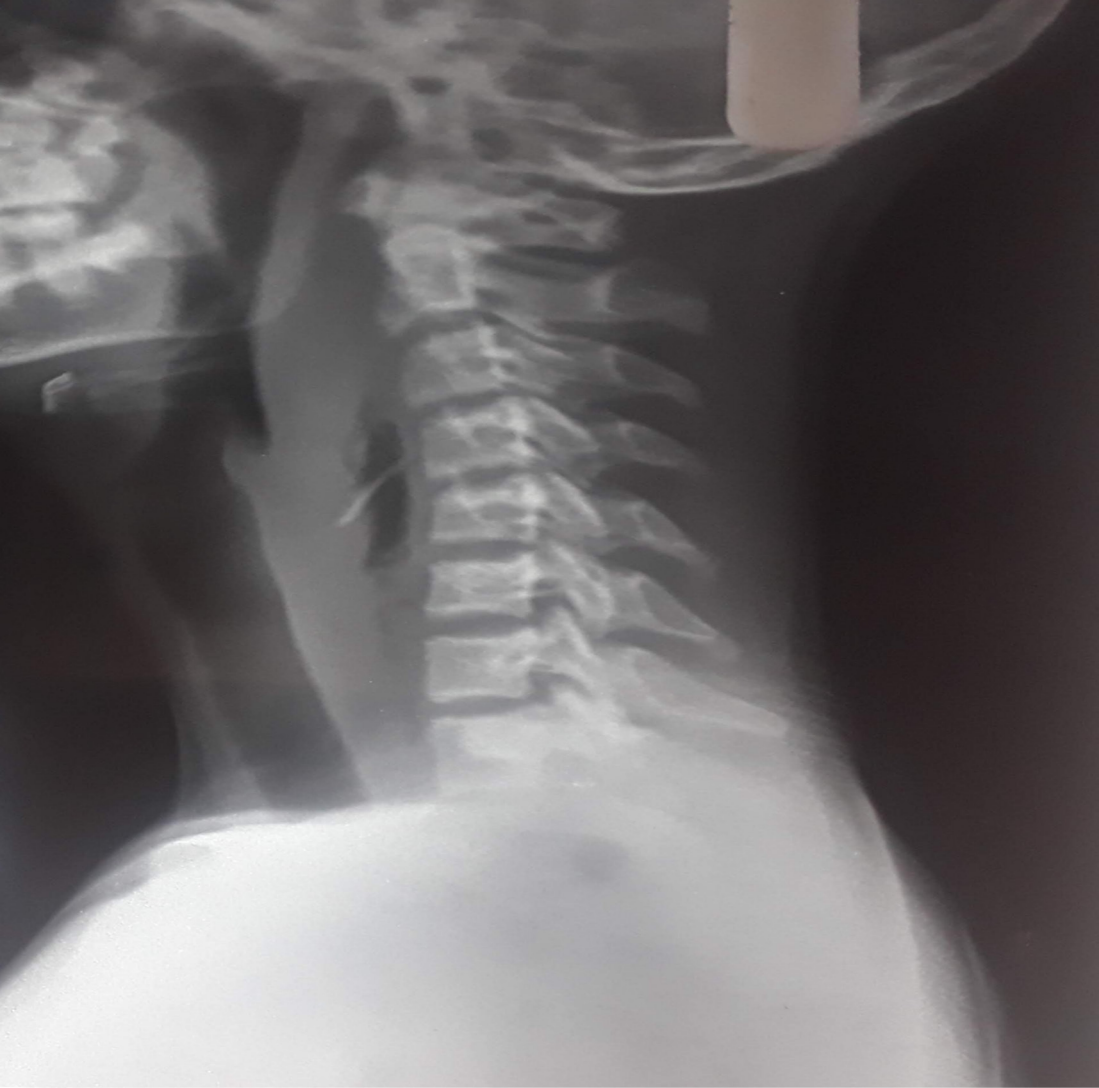
X‐ray soft tissue neck and lateral view showing radio opaque shadow at C3‐C5 level.

**FIGURE 2:**
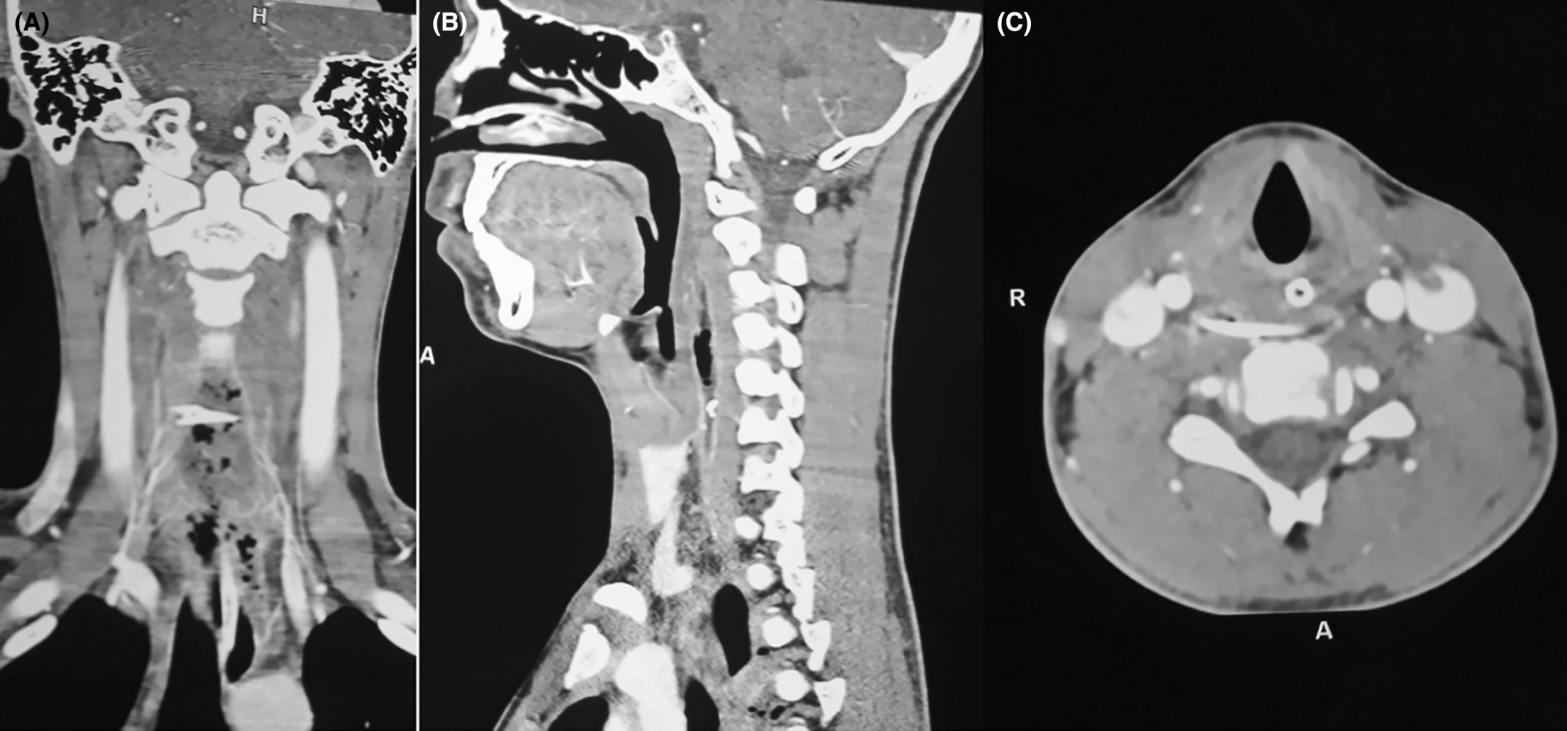
(A) CT coronal‐linear horizontally oriented bone density shadow; (B) Ct sagittal vertically elongated hypodense area in front of C3‐C6 and hyperdense shadow in front of C5‐C6; (C) CT axial horizontally placed foreign body at C5.

**FIGURE 3:**
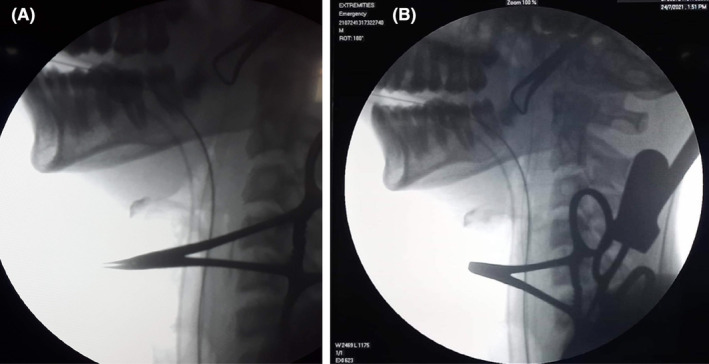
(A) C‐arm pre removal of the foreign body shows linear radio‐opaque shadow in front of C3‐C5 level; (B) C‐arm post removal of the foreign body shows no shadow.

**FIGURE 4:**
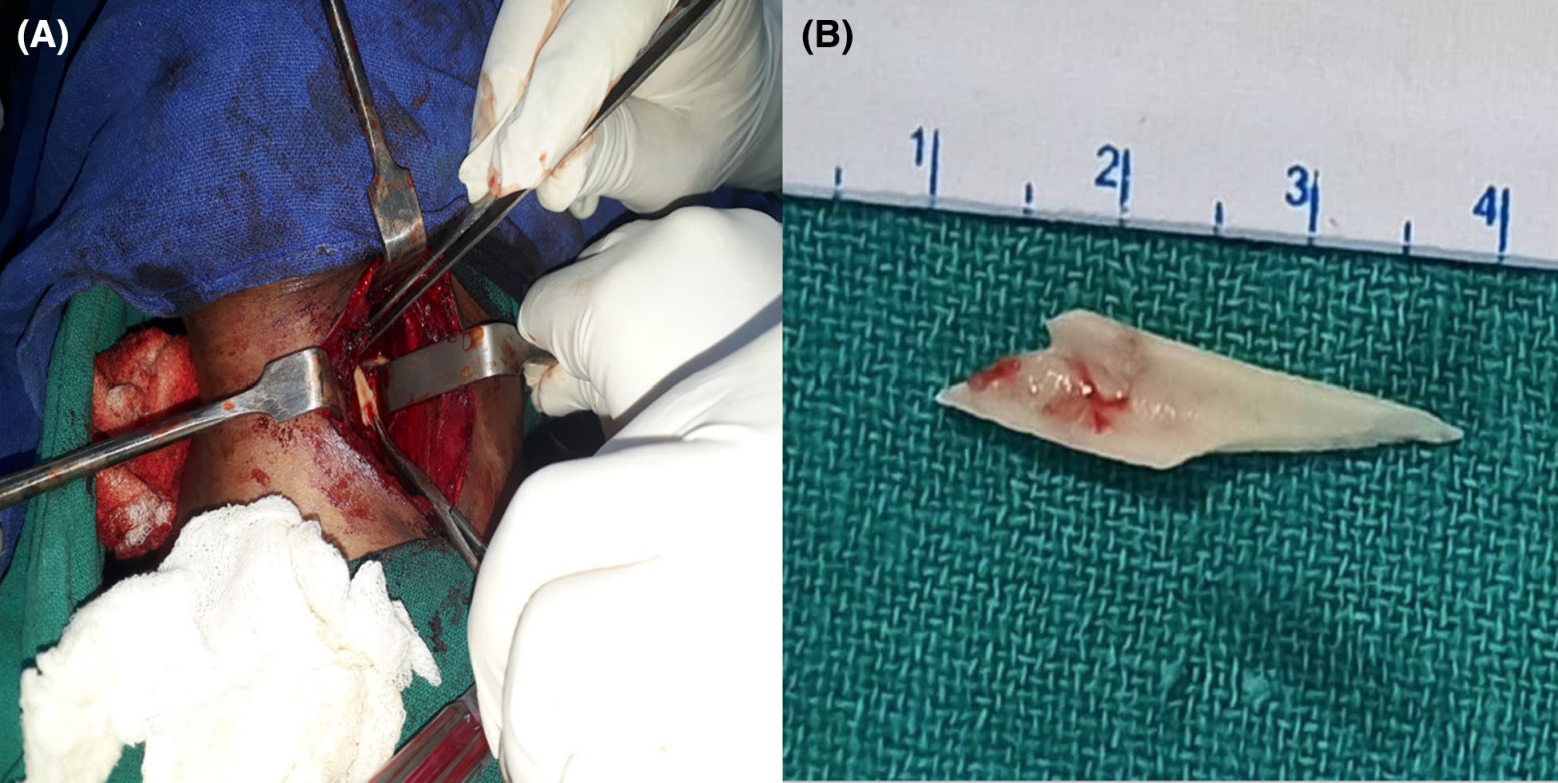
(A) Intraoperative visualization of Foreign body; (B) Extracted Foreign body(Bone).

X‐ray soft tissue neck lateral view showed a radiopaque shadow at the level of the C3‐C5 region (Figure 1). The patient underwent Rigid esophagoscopy for foreign body removal under general anesthesia. However, foreign bodies could not be seen in the pharynx and esophagus. Only pus came out from the left pyriform fossae area which was drained via suction. The procedure was abandoned and the patient was shifted to the recovery ward. The repeat X‐ray on the next day showed a similar shadow at the same location. To localize the exact site of the foreign body, a CT‐scan was done (Figure 2). Finally, the foreign body was removed via transcervical approach. A transverse horizontal incision was given on the left side at the level of the upper border of the thyroid cartilage. Sternocleidomastoid was retracted posteriorly and a great vessel anteriorly that led to the retropharyngeal space. It was very difficult to localize foreign bodies even by intraoperative palpation. C‐arm was used to localize the foreign body, which was present outside the lumen of the esophagus and was removed (Figures 3 and 4). The wound was cleaned thoroughly, and the drain was kept. It was closed in layers. Postoperatively, the condition was uneventful. NG feeding was done till the 10th postoperative day (POD), then oral feeding was resumed and the child was discharged on the 12th POD.

## 3 DISCUSSION

We did an X‐ray soft tissue neck which clearly visualized a radiopaque shadow and a radiolucent area suggestive of a foreign body with a retropharyngeal abscess. Plain X‐ray is very specific which showed air in the retropharyngeal abscess.^6^ We started intravenous empiric antibiotics therapy which prevents mediastinitis. It is important to perform rigid esophagoscopy and foreign body removal. In this case, only pus came out from the left pyriform fossa area which we suck out; however, no foreign body was found. A similar study was found by Thakur et al. who opened the neck to remove the foreign body after failed endoscopic removal.^7^

It is important to reassess and find the exact site of the foreign body. So, we performed a CT scan of the neck and thorax to visualize the exact location of the foreign body. However, it was difficult to distinguish luminal or extraluminal foreign bodies by radiological imaging. Decisions should be taken to remove foreign bodies by an alternative approach if imaging confirms the exact location. We decided to remove the foreign body by transcervical approach. Localization is difficult in the retropharyngeal space. So, the use of an intraoperative C‐arm guides the surgeon to locate the foreign body and remove it.

Foreign body impaction in the esophagus is a common pediatric emergency condition.^3^ Objects with smooth surfaces like marbles, batteries, coins, toys, magnets, bone, plastic candies are commonly ingested by children, and coin is the most common foreign body which accounts for 80% of all foreign bodies.^4^ The common site of foreign body impaction is the cricopharyngeal junction, the aortic area, and the gastroesophageal junction. Out of these, the cricopharyngeal junction is the commonest of all.

The clinical presentation depends on the site, size, shape, and types of the objects ingested. Patients commonly present with difficulty swallowing, odynophagia, drooling of saliva, throat discomfort, hoarseness, and rarely airway obstruction.

The sharp foreign bodies have a tendency to erode the mucosa or muscular layer of the esophagus causing mucosal edema which leads to mucosal ulceration, inflammation, and development of retropharyngeal abscess, mediastinitis, and perforation. It can develop serious life‐threatening conditions if it migrates into adjacent structures like the trachea, aorta, and mediastinum.^4^ Delay in diagnosis may even lead to septic shock and death. Our patient developed retropharyngeal abscess and respiratory difficulty.

All patients who presented with a history of foreign body ingestion should undergo evaluation with X‐ray soft tissue of the neck, the esophagus and the abdomen. Depending on the stability and clinician's opinion, further investigations like CT‐scan and direct visualization via rigid endoscopy may be needed for the removal of the foreign bodies.^5^

Management of the upper aerodigestive tract foreign bodies was described by Jackson and Jackson in 1937.^8^ The success rate of rigid instruments ranges between 94% and 100%. The incidence of esophageal perforation with a rigid endoscope is 0.34% with a 0.05% mortality rate.^9^ Loh ZM et al.^10^ have described the ingested foreign body which migrated extraluminal, although rare in occurrence is fraught with the potential to cause life‐threatening complications.

Considering high rates of successful removal of impacted foreign bodies, endoscopic retrieval almost always remains the preferred option of the management. However in some cases, like the one we have described above, where the foreign body lies in extramural space and cannot be seen via rigid esophagoscopy, we recommend a transcervical approach and the use of a C‐arm intraoperatively for the exact localization and removal of the foreign body. We think the decision of an open approach is much safer for the patient as compared to multiple endoscopic attempts that lead to significant complications.

## 4 CONCLUSION

The foreign body in the esophagus is a common otorhinolaryngological emergency. Most of the bony foreign body is commonly visualized by X‐rays and can be retrieved by rigid esophagoscopy. However, in some cases with negative findings on rigid esophagoscopy, it is important to reassess and localize the foreign body with the help of radiological imaging. Hence, we recommend removal of the foreign body by the open transcervical approach when rigid esophagoscopy fails.

## AUTHOR CONTRIBUTIONS

**Brihaspati Sigdel:** Conceptualization; data curation; funding acquisition; investigation; methodology; validation; writing – original draft; writing – review and editing. **Amrit Pokhrel:** Methodology; supervision; visualization. **Bipin Subedi:** Conceptualization; formal analysis; investigation; methodology. **Indra Subedi:** Conceptualization; investigation; methodology. **Bidhan Ghimire:** Conceptualization; data curation; funding acquisition; investigation. **Shankar Paudel:** Conceptualization; formal analysis; investigation; methodology.

## FUNDING INFORMATION

None.

## CONFLICT OF INTEREST STATEMENT

None.

## CONSENT

Written informed consent was obtained from the patient to publish this report in accordance with the journal's patient consent policy.

## Data Availability

Data available on request from the authors.
